# Historical Evolution of Terminology and Current Classification of Neuroendocrine Neoplasms of the Larynx and Mixed Neuroendocrine–Non-Neuroendocrine Neoplasms (MiNENs): A Narrative Review †

**DOI:** 10.3390/diagnostics16172829

**Published:** 2026-09-02

**Authors:** Martina Bradová, Abbas Agaimy, Alfio Ferlito

**Affiliations:** 1 Department of Pathology, Faculty of Medicine in Pilsen and Faculty Hospital, Charles University, 30599 Pilsen, Czech Republic; 2 Bioptic Laboratory, Ltd., 30599 Pilsen, Czech Republic; 3 Institute of Pathology, Erlangen University Hospital, Friedrich Alexander University of Erlangen-Nuremberg, 91054 Erlangen, Germany; abbas.agaimy@uk-erlangen.de; 4 Comprehensive Cancer Center, European Metropolitan Area Erlangen-Nuremberg (CCC ER-EMN), Friedrich Alexander University of Erlangen-Nuremberg, 91054 Erlangen, Germany; 5 Coordinator of the International Head and Neck Scientific Group, 35030 Padua, Italy; profalfioferlito@gmail.com

**Keywords:** terminology, classification, larynx, neuroendocrine neoplasms, MiNEN

## Abstract

This is a narrative review that describes the evolution of terminology and classification schemes of neuroendocrine tumors (NETs), neuroendocrine carcinomas (NECs), mixed neuroendocrine–non-neuroendocrine neoplasms (MiNENs), and paragangliomas of the larynx, with the contribution of immunohistochemistry and treatment of neuroendocrine neoplasms of the larynx. Neuroendocrine neoplasms of the larynx comprise both epithelial (NET and NEC) and neural crest-derived (paraganglioma) neoplasms. Their terminology has evolved substantially over time, with current classifications emphasizing biologically and clinically meaningful categories aligned with contemporary WHO frameworks of other organs. These neoplasms may show overlapping clinical presentation and histomorphological features, which can complicate accurate subclassification. However, precise classification is essential, as these entities display markedly different biological behavior, ranging from indolent to highly aggressive with poor prognosis, and their treatment is essentially histology-tailored. Neuroendocrine neoplasms are classified into three categories comprising six tumor subtypes: well-differentiated neuroendocrine tumors (NETs; grades 1, 2, and 3), poorly differentiated neuroendocrine carcinomas (NECs; small-cell and large-cell types), and paragangliomas. An additional, not yet WHO-recognized category is MiNENs (mixed neuroendocrine–non-neuroendocrine neoplasms), defined by the coexistence of neuroendocrine and non-neuroendocrine components. These tumors exhibit distinct biological behavior and clinical significance.

## 1. Introduction

Laryngeal neuroendocrine neoplasms (L-NENs) are very rare tumors representing much less than 1% of all laryngeal malignancies [1,2] and mostly appearing in the supraglottic area [3]. However, they represent the most common non-squamous-cell neoplasm presenting at this site [4,5,6,7,8]. L-NENs are separated into epithelial (neuroendocrine tumors [NETs] and neuroendocrine carcinomas [NECs]) and neural (paragangliomas) categories [2,8,9,10]. Mixed neuroendocrine–non-neuroendocrine neoplasms (MiNENs) also belong to the epithelial category but with morphologically and immunohistochemically distinct neuroendocrine and non-neuroendocrine components [11].

## 2. Historical Connotations of Epithelial Neuroendocrine Neoplasms

Over time the preferred terminology for the epithelial group has evolved and many approaches have been established for treatment strategies and clinical guidelines, with the most preferred terminology adopting the recommendations of the lung classification approach [12]. The historical evolution of classification of L-NEN is depicted in Table 1.

From a historical perspective, the recognition and classification of individual tumor types have evolved gradually and were often complicated by limited or unclear histopathologic criteria. Atypical carcinoid appears to have been described earliest, with Goldman et al. reporting a single case in 1969 [13]. Shortly thereafter, Olofsson and van Nostrand documented the first case of small-cell neuroendocrine carcinoma of the larynx in 1972 [14].

The identification of typical carcinoid tumors proved more problematic. Although Markel et al. reported what was initially considered the first case in 1980 [15], subsequent reevaluation by Woodruff and Ferlito demonstrated features more consistent with atypical carcinoid [9]. In the same year, Gehanno et al. described another case labeled as typical carcinoid; however, insufficient histologic documentation makes definitive classification uncertain [16]. The first well-substantiated case of typical carcinoid was ultimately reported by Duvall et al. in 1983 [17].

Historically, the terminology used for neuroendocrine neoplasms (NENs) has been inconsistent and often confusing, encompassing a broad spectrum of tumors, including both indolent lesions and clearly malignant entities such as small-cell and large-cell neuroendocrine carcinoma [18,19]. An early attempt at standardization was made by Gould et al. in 1982, who proposed a classification based on the degree of differentiation [20]. In this system, typical carcinoid was designated as very well-differentiated neuroendocrine carcinoma, atypical carcinoid as well-differentiated neuroendocrine carcinoma, and small-cell (undifferentiated) carcinoma as intermediate to poorly differentiated neuroendocrine carcinoma. In 1985, Woodruff et al. proposed a classification that included typical carcinoid and a category of neuroendocrine carcinoma (NEC) with two subtypes: small-cell NEC, which resembles its pulmonary counterpart, and large-cell (LC) NEC, characterized by an organoid pattern of cells with non-small-cell morphology [21]. It is noteworthy that this laryngeal large-cell NEC category was proposed 6 years before formal diagnostic criteria for pulmonary large-cell NEC were established [22].

Large-cell neuroendocrine carcinoma represents a more recent addition to the spectrum. While cases had been previously reported, clear diagnostic criteria specific to the larynx were not established until Greene et al. in 2005 [23], who proposed adopting criteria analogous to those used for pulmonary large-cell neuroendocrine carcinoma [24]. It is likely that some earlier tumors classified as atypical carcinoid would meet current criteria for large-cell neuroendocrine carcinoma, making the true first description difficult to ascertain.

The classification of pulmonary NEC [25] was adopted for laryngeal NEC as an alternative terminological approach to better reflect histologic features and biological behavior. In this system, as described by Wenig et al., tumors were categorized into well-differentiated, moderately differentiated, and poorly differentiated NECs [26]. The 1991 WHO classification adopted a similar framework but used different terminology, grouping tumors into typical carcinoid, atypical carcinoid, and neuroendocrine carcinoma (with small-cell, intermediate-cell, and large-cell subtypes).

A major step toward standardization came with the WHO classification of head and neck tumors in 2005 [27]. This system defined the principal categories as typical carcinoid, atypical carcinoid, small-cell neuroendocrine carcinoma, combined small-cell carcinoma with non-small-cell components, and paraganglioma. In parallel, these entities were aligned with a grading framework: typical carcinoid as well-differentiated (grade I), atypical carcinoid as moderately differentiated (grade II), and small-cell neuroendocrine carcinoma as poorly differentiated (grade III). Although large-cell neuroendocrine carcinoma was acknowledged, it was not formally separated and was generally subsumed under atypical carcinoid.

In the updated WHO Classification of Head and Neck Tumours of 2017, the categories of moderately and poorly differentiated neuroendocrine carcinomas were further refined. The moderately differentiated group was restricted to atypical carcinoid (grade II) [28], while the poorly differentiated category was subdivided into small-cell and large-cell neuroendocrine carcinomas (grade III) [29].

The most recent 2022 WHO Classification of Head and Neck Tumours (fifth edition) introduced a significant structural change, whereby tumor types shared across multiple organ systems are grouped together into a separate section rather than described individually within each anatomical site [30]. NENs are therefore separated into ectopic or invasive pituitary neuroendocrine tumors (PitNETs)/pituitary adenoma (which is not a part of this overview) and neuroendocrine tumors (NETs) that are defined as well-differentiated epithelial neuroendocrine neoplasms and are stratified into three grades according to their proliferative activity. In contrast to gastroenteropancreatic tract NETs, the category of grade 3 (G3) head and neck NETs is currently still considered provisional in the 2022 WHO Classification for Head and Neck Tumours, and its definitive status awaits further validation.

A Ki-67 proliferation index of 20% was applied to separate NET G1/G2 (<20%) from the provisional NET G3 category (>20%). It is important to emphasize that the diagnosis of NET G3 requires the presence of well-differentiated morphology; a high Ki-67 index alone in a tumor with poorly differentiated morphology would warrant classification as NEC, not NET G3. For the distinction between G1 and G2 tumors, the WHO does not provide a definitive Ki-67 cut-off, and grading relies primarily on mitotic count and the presence of necrosis (Table 2).

**Table 1:** Historical evolution of the classification systems for laryngeal neuroendocrine neoplasms.

Classic System	Gould 1983 [25]	Woodruff et al. 1985 [21]	Wenig et al. 1988 [26]	Ferlito and Friedmann 1989	Shanmugaratnam (WHO 1991) [22]	Wick 2000	Mills et al. 2000	Mills 2002	Barnes et al. (WHO. 2005) [27]	Brandwein- Gensler et al. 2009	El Naggar et al. (WHO 2017) [29]	WHO (5th Edition. 2022) [30]
Carcinoid	Very well-differentiated neuroendocrine carcinoma	Typical carcinoid	Well-differentiated neuroendocrine carcinoma	Typical carcinoid	Carcinoid tumor (typical carcinoid tumor)	Grade I neuroendocrine carcinoma	Carcinoid	Well-differentiated neuroendocrine carcinoma	Typical carcinoid	Well-differentiated neuroendocrine carcinoma	Well-differentiated neuroendocrine carcinoma (typical carcinoid, Grade I)	NET (Well-differentiated neuroendocrine tumor) Grade 1 Grade 2 Grade 3
Atypical carcinoid	Well-differentiated neuroendocrine carcinoma	Large-cell neuroendocrine carcinoma	Moderately differentiated neuroendocrine carcinoma	Atypical carcinoid	Atypical carcinoid tumor	Grade II neuroendocrine carcinoma	Moderately differentiated neuroendocrine carcinoma	Moderately differentiated neuroendocrine carcinoma	Atypical carcinoid	Moderately differentiated neuroendocrine carcinoma	Moderately differentiated neuroendocrine carcinoma (atypical carcinoid, Grade II)	Neuroendocrine carcinoma Small-cell neuroendocrine carcinoma Large-cell neuroendocrine carcinoma Merkel-cell carcinoma
Small-cell undifferentiated carcinoma	Intermediate/poorly differentiated neuroendocrine carcinoma	Small-cell neuroendocrine carcinoma	Poorly differentiated neuroendocrine carcinoma	Small-cell neuroendocrine carcinoma	Small-cell carcinoma (small-cell neuroendocrine carcinoma)	Grade III neuroendocrine carcinoma	Small-cell neuroendocrine carcinoma	Poorly differentiated neuroendocrine carcinoma (small-cell and large-cell subtypes)	Small-cell carcinoma, neuroendocrine type	Poorly differentiated neuroendocrine carcinoma	Poorly differentiated neuroendocrine carcinoma (subgroups: small-cell carcinoma, Grade III and large-cell neuroendocrine carcinoma Grade III)	
									Combined small-cell carcinoma, neuroendocrine type, with non-small-cell carcinoma			Mixed neuroendocrine–non-neuroendocrine neoplasms (provisional-emerging entity)
										Large-cell undifferentiated neuroendocrine carcinoma		
Paraganglioma	Paraganglioma	Paraganglioma	Paraganglioma	Paraganglioma	Paraganglioma	Paraganglioma	Paraganglioma	Paraganglioma	Paraganglioma	Paraganglioma	Paraganglioma	Paraganglioma

Adapted from Ferlito et al. PMID: 19536850 https://doi.org/10.1002/hed.21162 (accessed on 29 August 2026) [10].

**Table 2:** WHO Classification of Head and Neck Tumours (fifth edition), 2022—groups of neuroendocrine neoplasms. Adapted from the WHO Classification of Head and Neck Tumours (5th edition).

Neuroendocrine Neoplasm (NEN)	Tumor Category	Diagnostic Criteria
Well-differentiated NEN (NET)	Well-differentiated NET, grade 1 (G1 NET)	No necrosis and <2 mitoses/2 mm^2^ Ki-67 < 20% *
	Well-differentiated NET, grade 2 (G2 NET)	Necrosis and/or 2–10 mitoses/2 mm^2^ Ki-67 < 20% *
	Well-differentiated NET, grade 3 (G3 NET) *	>10 mitoses/2 mm^2^ Ki-67 > 20% * Absence of NEC cytomorphology
Poorly differentiated NEN (NEC)	Small-cell NEC	>10 mitoses/2 mm^2^ Ki-67 > 20% (often >70%) Small-cell NEC cytomorphology ^a^
	Large-cell NEC	>10 mitoses/2 mm^2^ Ki-67 > 20% (often >50%) Large-cell NEC cytomorphology ^b^

NET—neuroendocrine tumor; NEC—neuroendocrine carcinoma. * Provisional criteria applied. ^a^ Small-cell neuroendocrine carcinoma is characterized by scant cytoplasm and densely hyperchromatic nuclei with frequent nuclear molding. The chromatin is finely granular, nucleoli are typically inconspicuous, and the cells are small—generally not exceeding the size of approximately three lymphocytes. Apoptotic bodies are commonly observed, often accompanied by extensive necrosis. ^b^ In contrast, large-cell neuroendocrine carcinoma shows a more abundant cytoplasm and tends to grow in nested, organoid, or trabecular patterns. The nuclei are larger and round, with clearly visible nucleoli. Additional architectural features may include peripheral palisading and rosette-like structures, while comedonecrosis is frequently present. Ki-67 index of 20% separates NET G1/G2 from the provisional NET G3 category. Diagnosis of NET G3 requires well-differentiated morphology. No validated Ki-67 cut-off exists for the distinction between G1 and G2. NEC is diagnosed primarily on morphology; a high Ki-67 index is a supportive but not defining feature.

The second subgroup in the recent WHO classification comprises poorly differentiated neuroendocrine neoplasms (NECs), which are further divided into small-cell and large-cell NEC and Merkel-cell carcinoma. Both small-cell and large-cell NECs are characterized by poor histological differentiation (lack of characteristic organoid NET pattern) and high proliferative activity, defined by more than 10 mitoses per 2 mm^2^ and a Ki-67 index usually exceeding 50%. Poorly differentiated NECs (small-cell and large-cell types) are characterized by a very high proliferation rate, with Ki-67 indices typically exceeding 70–80%. Small-cell NEC typically shows very high Ki-67 values (often >70%) and a corresponding small-cell morphology, whereas large-cell NEC usually demonstrates slightly lower, though still elevated, Ki-67 levels (commonly >50%) and is defined by large-cell cytological features. However, it must be stressed that the diagnosis of NEC is fundamentally based on morphological criteria—including nuclear molding, abundant necrosis, and a high nuclear-to-cytoplasmic ratio—and is not made on the Ki-67 index alone. The Ki-67 index serves as a supportive, not definitive, diagnostic tool in this context.

## 3. Historical Connotations of Neural Neuroendocrine Neoplasms (Paragangliomas)

Paraganglioma of the larynx was first described by Blanchard and Saunders in 1955 under the term “chemodectoma” [31]. Subsequent cases were reported under the term “glomus tumor” (nonchromaffin paraganglioma) of the larynx; however, many of these were not well histologically defined, were diagnostically questionable, and most likely represent well-differentiated NEN, G2 (atypical carcinoid tumors) or G1 (typical carcinoid tumors) or even poorly differentiated NEC (small-cell NEC) [32,33,34].

## 4. Biomarkers and Immunohistochemistry 

### 4.1. Epithelial Laryngeal Neuroendocrine Neoplasms: Detailed Biomarker List

For epithelial laryngeal NENs, besides clinical findings and tumor cytomorphology, there are usually three immunohistochemical (IHC) steps toward the diagnosis including: (1) confirmation of epithelial origin; (2) confirmation of neuroendocrine differentiation; and (3) assessment of grade/aggressivity of the disease, specifically for the NEC group [35]. And two additional steps may be required to help guide the clinical management of patients with NENs including (4) site-specific markers, along with (5) predictive and prognostic biomarkers. But these two points are beyond the scope of this paper, will be discussed briefly, and have been reported elsewhere [35,36]. Pathology of laryngeal epithelial tumors may overlap with other entities (e.g., paraganglioma, metastasis from non-laryngeal origin, SWI/SNF-deficient neoplasms of different areas, etc.) [37,38,39]; the approach to the disease remains in clinico-pathological correlation, especially in advanced disease (Table 3). 

***(1) Confirmation of epithelial origin:***

For the epithelial origin, the commonly used markers include pan-cytokeratins (AE1/AE3) [6] or low-molecular weight cytokeratins (CK8/18) [37,40]. These markers are generally expected to be positive in NEN. EMA and CK7 may be used as supportive IHC markers [41]. Clinically and diagnostically, the key conceptual value of keratin positivity is that it helps distinguish epithelial NENs from laryngeal paraganglioma, which is typically keratin-negative [42]. CK20 can be expressed in a subset of head and neck laryngeal NENs, but it is not a consistent diagnostic hallmark [2]. Any CK20 positivity in a particular histological background should prompt differentiation from Merkel-cell carcinoma and metastases from digestive tract NEN [43]. EpCAM (epithelial cell adhesion molecule) is a transmembrane glycoprotein that serves as a reliable marker of epithelial differentiation. In the differential diagnosis of laryngeal NENs, EpCAM positivity supports an epithelial origin, thereby helping to distinguish epithelial NETs and NECs from paraganglioma, which lacks EpCAM expression [44].

***(2) Confirmation of neuroendocrine differentiation:***

Neuroendocrine differentiation is mostly recognizable on histology (organoid pattern with a diversity of arrangement, pepper–salt chromatin, and purple to pale-eosinophilic cytoplasm) and can be confirmed using a combination of highly sensitive and relatively more specific markers. In routine pathology, the most widely applied markers are synaptophysin and chromogranin A [6,35,45,46,47], with the former being highly sensitive and the latter being both sensitive and highly specific for NENs. Many laryngeal epithelial NENs show positivity for one or both; therefore, using them together is common practice. A major modern second-generation neuroendocrine IHC marker with high sensitivity and acceptable specificity is the transcriptional repressor insulinoma-associated protein 1 (INSM1), a nuclear marker that has shown strong diagnostic utility across neuroendocrine tumor categories [6]. In many practical panels, INSM1 + synaptophysin (±chromogranin A) provides a highly effective approach to confirm neuroendocrine differentiation. 

In the differential diagnosis of laryngeal NENs, two additional entities of non-laryngeal origin should be considered: medullary thyroid carcinoma and parathyroid tumors. Both can present as neck masses and may involve the larynx by direct extension or metastasis. Medullary thyroid carcinoma is characterized by expression of calcitonin and TTF-1. Importantly, a subset of laryngeal epithelial NENs may also express calcitonin and can therefore mimic medullary thyroid carcinom [6]. Because of this overlap, calcitonin should always be interpreted in context, including clinical history, imaging findings, and additional thyroid-related workup. Parathyroid tumors express parathyroid hormone (PTH) and GATA3, and represent a particular diagnostic pitfall, as they can be GATA3-positive and cytokeratin-negative, which may lead to misclassification as paraganglioma [48,49]. Therefore, in the appropriate clinical and anatomical context, PTH and calcitonin immunohistochemistry should be included in the diagnostic panel [49].

***(3) Assessment of grade/aggressivity of the disease specifically for NEC group***

Mitotic count is usually sufficient to distinguish between categories, particularly among well-differentiated NETs, and together with the presence of necrosis and the characteristic histological tumor profile in larger surgical specimens forms the basis of NET stratification. However, in small or crushed specimens, Ki-67 proliferative activity serves as a well-established adjunct, helping to resolve doubtful cases and avoid overdiagnosis, especially when differentiating NET G3 from NEC [6,19,28]. Overall, Ki-67 remains an indispensable adjunct in the diagnostic workup of laryngeal NENs, but its role must be carefully aligned with the current WHO diagnostic criteria:

For high-grade tumors (especially NECs), additional markers are often used to support the biological phenotype: p53 and Rb1 (Retinoblastoma 1) [6]. Abnormal p53 expression (e.g., overexpression or null pattern) and loss of Rb1 are commonly associated with higher-grade neuroendocrine carcinoma biology and can strengthen classification when morphology is suggestive [6].

***(4) Site specific markers***

Given the therapeutic implications, a diagnosis of a well-differentiated NET often warrants additional immunohistochemical workup to determine the site of origin, particularly when this information is not clear to the treating clinician. In contrast, establishing the primary site in a poorly differentiated NEC is less frequently pursued, as these tumors tend to share similar biological behavior and are managed with largely uniform treatment approaches [35,50,51].

A critical caveat in the interpretation of these transcription factors is that they may be promiscuously and aberrantly expressed in poorly differentiated neuroendocrine carcinomas (NECs) regardless of the primary tumor site. Therefore, their lineage specificity is significantly reduced in high-grade neoplasms, and results should be interpreted with caution and in conjunction with the overall clinical and pathological context.

***(5) Predictive and prognostic biomarkers***

Additional biomarkers beyond the primary site and proliferation index may provide predictive and prognostic information in NETs, although most are assessed in surgical specimens and only selectively in biopsies; importantly, the majority have been described outside the larynx and have not been well validated in this location [35]. Somatostatin receptor (SSTR) expression, particularly SSTR2A, may guide eligibility for somatostatin analogs and peptide receptor radionuclide therapy, with loss of SSTR2A associated with worse surviva [52,53,54]. Hormonal marker expression is clinically relevant in functional NETs, both for identifying syndromes and enabling biochemical monitoring.

Markers such as loss of E-cadherin and β-catenin correlate with higher grade in pulmonary and gastrointestinal NETs and an increased potential for frequent lymph node metastasis [55,56], while in pancreatic NETs, expression of CEACAM1, CK19, and CD117 is linked to more aggressive behavior [57,58,59]. Markers tested for gastroenteropancreatic neuroendocrine tumors have not been well studied in L-NET [60,61]. Additional adverse prognostic markers include COX-2, p21, p18, and Rb [62,63] while hormonal subtyping (e.g., L-cell phenotype) may carry prognostic value in hindgut NETs [64].

### 4.2. Neural Laryngeal Neuroendocrine Neoplasms (Paragangliomas): Detailed Biomarker List

Laryngeal paragangliomas recapitulate their sympathetic and parasympathetic counterparts morphologically and immunohistochemically. Histologically, the classic pattern is zellballen architecture: nests of chief cells separated by a rich capillary network, with peripheral sustentacular cells. The chief cells usually have abundant pale to eosinophilic cytoplasm, round-to-oval nuclei with mild to moderate atypia, rare mitoses, and typically no necrosis. The lesion is often very vascular [30].

Immunohistochemical evaluation of laryngeal paraganglioma is aimed at confirming neuroendocrine differentiation, demonstrating the characteristic sustentacular cell population, and excluding epithelial neuroendocrine neoplasms, particularly through the absence of keratin expression. In addition, selected markers provide insight into tumor biology and hereditary background, especially within the SDH pathway (Table 4).

The chief (neuroendocrine) cells of paraganglioma typically show strong positivity for chromogranin A and synaptophysin, which represent the core neuroendocrine markers used in routine diagnostics [65]. INSM1 may also be positive and serves as a useful modern neuroendocrine marker, although it is not specific for paraganglioma and must be interpreted in conjunction with other findings [65]. In contrast to epithelial neuroendocrine tumors, paragangliomas are characteristically negative for cytokeratins (including AE1/AE3 and CAM5.2), a feature that is diagnostically crucial in distinguishing them from epithelial laryngeal NENs.

Sustentacular cells, which can be highlighted by S100 protein, SOX10, and GFAP, are a characteristic finding in paragangliomas, where they surround the classic zellballen architecture. SOX10 has emerged as a particularly useful and often more reliable marker of sustentacular (Schwannian) differentiation, especially in cases where S100 staining is weak or patchy [66]. GFAP may also label sustentacular cells, although its expression is less consistent and is generally considered supportive rather than essential [67]. However, the presence of sustentacular cells is not pathognomonic for paraganglioma, as they can also be found in NETs from various sites, including the larynx [68]. Conversely, sustentacular cells may be significantly reduced or absent in aggressive paragangliomas [69]. Therefore, sustentacular cell markers should not be used in isolation to distinguish between different NENs, and the diagnosis of paraganglioma must rely on the complete immunohistochemical profile, with cytokeratin negativity being the most critical finding to distinguish it from an epithelial NET.

Several additional markers can support the diagnosis in difficult cases. GATA3 is frequently expressed in paragangliomas and can be helpful in the differential diagnosis [65,70,71], while PHOX2B has been shown to be a sensitive and relatively specific marker for paraganglionic differentiation, making it a valuable addition to the diagnostic panel [70,71].

Markers related to catecholamine synthesis, such as tyrosine hydroxylase (TH), dopamine beta-hydroxylase (DBH), and phenylethanolamine N-methyltransferase (PNMT), may be expressed in paragangliomas and can support their neurochemical phenotype; however, their expression is variable and they are more often used in specialized or research settings [65]. Similarly, choline acetyltransferase (ChAT) has been described in head and neck paragangliomas but is not part of routine diagnostic panels in most laboratories [72].

A critical component of modern paraganglioma workup is evaluation of the succinate dehydrogenase (SDH) complex, particularly SDHB immunohistochemistry [73,74,75]. Loss of SDHB expression suggests an underlying SDHx mutation and is therefore an important screening tool with both diagnostic and genetic implications. SDHA immunostaining may also be informative, particularly in suspected SDHA-mutant tumors [76]. Additional markers such as alpha-inhibin and CAIX [77] have been explored in relation to molecular subgroups (e.g., “cluster 1” tumors), but they currently serve only as supportive or research biomarkers and cannot replace SDHB testing.

The proliferation index (Ki-67) can be reported in paragangliomas; however, it is important to emphasize that no single immunohistochemical marker reliably predicts malignant behavior in these tumors [65]. Therefore, Ki-67 should be interpreted as part of a broader clinicopathologic assessment rather than as a standalone prognostic indicator.

From a practical standpoint, a first-line immunohistochemical panel for suspected laryngeal paraganglioma typically includes chromogranin A, synaptophysin, S100, SOX10, SDHB, GATA3, PHOX2B, and Ki-67. The absence of cytokeratin expression remains a key diagnostic feature; if keratin positivity is identified, the diagnosis should be carefully reconsidered, including the possibility of an epithelial neuroendocrine neoplasm or an unusual variant of paraganglioma.

## 5. Mixed Neuroendocrine–Non-Neuroendocrine Neoplasm (MiNEN)

While the definition of a MiNEN has been standardized in the WHO Classifications of Endocrine and Neuroendocrine Tumours (5th edition) [78] and Digestive System Tumours (5th edition) [79] as a tumor consisting of at least 30% of both neuroendocrine and non-neuroendocrine components that differ histologically and immunohistochemically, it is important to note that this commonly cited 30% threshold is extrapolated from the gastrointestinal and pancreatic literature and has not been formally validated for laryngeal primaries. A specific cut-off for both components in the larynx has not yet been established [11], and the applicability of this threshold in the larynx therefore remains an open question. Consequently, any assumptions regarding the adequacy or inadequacy of this threshold in laryngeal MiNEN should be interpreted with caution given the limited larynx-specific evidence currently available [11].

## 6. Historical Connotations of MiNEN

Previously, the terminology varied, and this entity was referred to by different names, which led to confusion among pathologists and clinicians. The terminology included: mucin-producing carcinoid; composite carcinoid–adenocarcinoma; composite carcinoid tumor; collision tumor; (adeno)carcinoma with divergent differentiation; multidirectionally differentiated carcinoma; endocrine mucin-producing carcinoma; composite glandular–neuroendocrine mixed tumor; mixed adenocarcinoid tumor; composite glandular–endocrine tumor or carcinoma; scirrhous argyrophil cell carcinoma; mucinous carcinoid; signet ring cell carcinoid; adenocarcinoma ex goblet cell carcinoid; mixed exocrine–endocrine tumor; mixed exocrine–neuroendocrine tumor; or combined exocrine–neuroendocrine tumor or carcinoma [11]. Most of these terms were used in the context of digestive system tumors.

The penultimate terminology referred to these tumors as mixed adenoneuroendocrine carcinoma (MANEC) [80]. However, it was later recognized that the non-neuroendocrine component may be non-adenocarcinomatous; to reflect this variability, the terminology was revised to MiNEN.

## 7. Theory of MiNEN Origin 

The origin of MiNENs remains uncertain, and proposed models—such as collision of two components, a common precursor with divergent differentiation, or, in some contexts (e.g., thyroid), host/driver hypotheses—are speculative and not uniformly supported across organ systems [11,81,82,83,84,85]. Robust molecular evidence is currently limited outside certain sites (notably the urogenital tract) [86,87]. In colorectal MiNENs, some studies suggest a clonal, shared origin with a ‘common trunk’ of mutations across components, but these findings do not imply universal presence of specific driver alterations (e.g., APC, KRAS, SMAD4) in all MiNENs. Thus, while cross-organ comparisons can generate hypotheses, conclusions about MiNEN biology in laryngeal tumors should be framed as provisional and contingent on tumor-specific molecular data [86,87].

## 8. Laryngeal MiNEN 

MiNEN of the larynx has been reported in 25 cases in the English literature so far [88,89,90,91,92,93,94,95,96,97,98,99,100,101,102,103]. In 22 cases, the tumor consisted of a combination of squamous-cell carcinoma and small-cell carcinoma; in one case, the neuroendocrine component was atypical carcinoid combined with squamous-cell carcinoma [96]. In two cases, the neuroendocrine component was large-cell neuroendocrine carcinoma combined with squamous-cell carcinoma [100,101] Table 5. 

Twenty-three patients with laryngeal MiNEN were male and only two were female. The typical age distribution falls between the 5th and 6th decades of life, with a mean age of 56.2 years (age range 32–83 years). Most patients were current or former smokers.

Immunohistochemical characterization typically requires an appropriate panel that includes squamous markers (p63/p40, CK5/6) and neuroendocrine markers (synaptophysin, chromogranin A, and INSM1), along with assessment of the Ki-67 proliferation index for grading [11].

In cases where preoperative biopsies are performed, small sample size may limit diagnostic accuracy in MiNEN, as both tumor components must be present in the specimen [102]; consequently, the proportion of each component cannot be reliably quantified. Moreover, a larynx-specific cut-off defining the minimum proportion of each component has not yet been established.

Eight patients (32%) died from the disease after a mean follow-up of 15.4 months (range 1–48 months). Five patients (20%) were alive with the disease, with a mean follow-up of 21.6 months (range 3–44 months). Nine patients (36%) were disease-free at a mean follow-up of 23.1 months (range 6–77 months). In three patients, follow-up information was unavailable.

Most patients developed at least regional lymph node (neck) metastases (17 cases, 68%) and/or distant metastases (4 cases, 16%), which occurred predominantly in the bones (n = 4) and lungs (n = 3); one case each involved the brain and peritoneum. Importantly, among the four patients with distant metastases, all had concurrent locoregional involvement.

## 9. Clinical Behavior and Prognosis of Laryngeal MiNEN

Based on the histopathological features reported in most cases, where both components often blend with a continuous morphological spectrum, laryngeal MiNENs are likely to arise from divergent differentiation of a common progenitor cell. Although direct molecular evidence is still lacking, this concept is strongly supported by the overall morphological context.

Mixed neoplasms exhibit distinct clinicopathological behavior compared to their pure neuroendocrine or non-neuroendocrine counterparts. Importantly, across different anatomical sites, they share key biological features that point toward a unified pathogenesis. Available molecular data, albeit limited, support potential common clonal origin of both components, indicating a single neoplastic process with divergent phenotypic expression [106].

MiNENs may thus be interpreted as an epiphenomenon of tumor heterogeneity, with serious implications for clinical behavior as well as for mechanisms of response and resistance to antineoplastic therapy [107]. In particular, when a high-grade neuroendocrine component—especially small-cell carcinoma—is present, it appears to drive tumor biology and dictate prognosis. These tumors can be best understood as the result of progression of a non-neuroendocrine neoplasm toward a more aggressive phenotype through the emergence and selection of highly malignant neuroendocrine clones.

In this context, the mixed morphology should not be viewed as a mere coexistence of two components, but rather as a reflection of clonal selection within a heterogeneous tumor population. This model explains the consistently poor clinical outcomes and underscores a critical diagnostic implication: even minimal foci of small-cell/neuroendocrine carcinoma are of major clinical significance and must not be overlooked. Consequently, the traditional requirement of a 30% threshold for each component to define MiNEN is likely inadequate and should be reconsidered, as it risks underestimating clinically decisive aggressive subclones [107]. In each case, both components should be quantified, defined by the most recently used terminology, and graded.

## 10. Treatment Considerations

The management of laryngeal NENs is strictly dependent on the histological subtype and tumor stage, requiring a multidisciplinary approach. A detailed discussion of therapeutic protocols is beyond the scope of this review and will be discussed in the particular ancilallary reviews. General principles can be outlined.

- Well-differentiated NETs (G1/G2): These are generally treated with complete surgical excision as the primary modality. Given their more indolent behavior, the role of adjuvant therapies is limited and not well-established.
- Poorly differentiated NECs (small-cell and large-cell): These are highly aggressive malignancies that are treated similarly to small-cell lung cancer. The mainstay of treatment is systemic chemotherapy, often combined with radiotherapy. Surgery may play a role in very early-stage disease or as a salvage procedure.
- MiNEN: Treatment should be directed at the most aggressive component, which is usually the neuroendocrine carcinoma or the poorly differentiated non-neuroendocrine component. Therefore, these tumors are often managed with chemoradiation protocols similar to those for high-grade NECs, with surgery reserved for selected cases.

## 11. Summarization of Practical Diagnostic Algorithm and Clinical Implications

Given the overlapping morphology of laryngeal NENs, a stepwise diagnostic approach is recommended. The initial assessment should be based on standard hematoxylin and eosin (H&E) staining to evaluate tumor architecture, cytological features, and the presence of necrosis. This morphological impression then guides a focused immunohistochemistry panel. The first-line markers should include pan-cytokeratin (e.g., AE1/AE3) and a cocktail of neuroendocrine markers, specifically synaptophysin, chromogranin A, and INSM1. Ki-67 is indispensable for grading and subclassification. This core panel allows for the initial separation into the main diagnostic categories:

- NET vs. NEC: Well-differentiated NETs (G1/G2) show organoid architecture with mild to moderate atypia and a Ki-67 index < 20%, while NECs display diffuse sheets of highly atypical cells with abundant necrosis and a very high Ki-67 index (typically >70%).
- NET vs. Paraganglioma: Both can show a nested (zellballen) pattern. The key discriminator is cytokeratin: NETs are strongly and diffusely positive for pan-cytokeratin, whereas paragangliomas are negative. S100 protein can highlight sustentacular cells at the periphery of nests in paraganglioma, but this finding is not specific.
- Diagnosis of MiNEN: This requires the morphological and immunohistochemical identification of two distinct components—one neuroendocrine (meeting criteria for NET or NEC) and one non-neuroendocrine (most commonly squamous-cell carcinoma). The MiNEN diagnosis should not be based on scattered neuroendocrine cells within a non-neuroendocrine tumor.

The clinical implications of accurate classification are profound, as treatment is histology-tailored and prognosis varies dramatically. For example, a well-differentiated NET G1 may be treated with conservative surgery, while a small-cell NEC requires urgent systemic chemotherapy. Given the complexity and rarity of these tumors, expert pathological review is strongly recommended to ensure diagnostic accuracy and guide appropriate clinical management.

Given the diagnostic complexity of rare head and neck cancers and the presence of unusual or mixed histologies such as laryngeal MiNEN, expert pathological review by a dedicated head and neck pathologist should be considered as a routine step in complex cases. The recent national survey and retrospective study by Filippini et al. [108] underscores the value of second opinions in refining diagnoses and guiding treatment, with major diagnostic discrepancies identified in a substantial subset of reviewed cases. Their findings align with the need for close collaboration among pathologists and clinicians in high-volume centers, particularly when morphology is ambiguous and molecular profiling can influence management. In MiNEN-like scenarios, morphological assessment remains foundational, while Ki-67 and other markers serve as supportive elements within the current WHO criteria, not as sole determinants [30].

## 12. Conclusions

In summary, the classification of laryngeal neuroendocrine neoplasms has undergone a significant evolution, culminating in the current WHO framework that recognizes well-differentiated NETs (G1 and G2), poorly differentiated NECs (small-cell and large-cell types), and paraganglioma as distinct, established entities. This review has traced this historical trajectory and highlighted the critical role of immunohistochemistry in achieving accurate diagnosis.

It is essential to distinguish these established entities from provisional and emerging categories. NET G3 is currently a provisional category in the 2022 WHO classification for head and neck tumors, and its definitive status and clinical behavior await further validation through larger studies. Similarly, laryngeal MiNEN is not yet a WHO-recognized entity. Its definition, particularly the applicability of the 30% threshold extrapolated from other organ systems, and its pathogenesis remain open questions that require dedicated investigation.

Future research priorities should focus on several key areas. First, the clinical and molecular characterization of laryngeal NET G3 is needed to determine if it represents a distinct biological entity warranting separate classification and specific treatment guidelines. Second, multi-institutional studies on laryngeal MiNEN are crucial to define validated diagnostic criteria specific to this site, and establish optimal management strategies. Third, given the rarity and diagnostic complexity of these tumors, the role of centralized expert pathological review should be formally evaluated as a standard of care to ensure diagnostic accuracy and guide appropriate, histology-tailored therapy. Addressing these unresolved questions will be essential to refine the classification and improve outcomes for patients with these rare neoplasms.

## Figures and Tables

**Table 3:** Most useful IHC/biomarkers in epithelial laryngeal NENs.

Marker	Typical Pattern in Laryngeal Epithelial NENs	Main Diagnostic Value/Comments
Pan-cytokeratins (AE1/AE3) ^R^	Regularly positive (diffuse)	Confirms epithelial lineage. Helpful to separate epithelial NEN from paraganglioma.
CK8/18/low-molecular-weight keratin	Commonly positive in laryngeal carcinoid/NEC (diffuse).	Very useful in small biopsies.
CK7 ^S^	Can be positive in some laryngeal NENs/NECs.	Supportive but not specific.
EMA ^S^	Often positive in epithelial NENs.	Supports epithelial differentiation.
CEA ^S^	Often positive in laryngeal carcinoid/atypical carcinoid; may be focal to diffuse.	Helpful, but not specific because CEA can overlap with medullary thyroid carcinoma.
Synaptophysin ^R^	Regularly positive, diffuse, recommended	One of the best first-line neuroendocrine markers.
Chromogranin A ^R^	Regularly positive, diffuse, recommended	Often the most specific classical neuroendocrine marker.
INSM1 ^R^	Regularly positive, diffuse, nuclear marker, recommended	Excellent modern marker for neuroendocrine differentiation.
CD56 ^S^	Commonly used supportive marker, but non-specific, not recommended in GEP-NEN.	Sensitive but much less specific.
Calcitonin	Positive in a subset	Important because some laryngeal NENs can be calcitonin-secreting and mimic MTC
TTF-1	Can be focal in laryngeal NECs.	Do not use alone to assign a lung primary; focal positivity can occur in laryngeal primaries.
CK20	Can be positive in some head and neck NENs	Should be interpreted with caution, diagnosis of Merkel-cell carcinoma (metastasis) and metastatic intestinal NENs should be excluded
p53 ^S^	Aberrant overexpression/null pattern common in high-grade NECs.	Useful in high-grade NEC characterization.
Rb (RB1) ^S^	Loss is common in small-cell/large-cell NEC.	Strongly supports high-grade NEC biology when lost.
p16 ^S^	Can be diffusely positive in NEC, but is not a reliable surrogate for HPV in this setting.	Interpret with HPV testing, not alone.
PD-L1	Studied in laryngeal/pharyngeal NECs.	Research/potential therapeutic biomarker; not yet a core diagnostic marker.
Ki-67 ^R^	Strongly recommended for prognostic stratification; high in NEC, lower in well-differentiated NETs.	Essential proliferation marker, especially for grading and risk assessment.
p63/p40/CK5/6 ^S^	Usually negative or only very focal in laryngeal NECs; useful mainly to exclude squamous differentiation, help to highlight SCC in MiNEN.	Helps in differential diagnosis with SCC/MiNEN.

^R^—routine; ^S^—supportive. GEP-NEN—gastroenteropancreatic neuroendocrine neoplasm; MiNEN—mixed neuroendocrine–non-neuroendocrine neoplasms; MTC—medullary thyroid carcinoma; NEC—neuroendocrine carcinoma; NEN—neuroendocrine neoplasm; NET—neuroendocrine tumor; RB1—retinoblastoma1; SCC—squamous-cell carcinoma.

**Table 4:** Distinguishing laryngeal epithelial neuroendocrine neoplasm from paraganglioma.

Question	Biomarker Clue	Interpretation
Is it epithelial?	AE1/AE3, CK7, EMA, CEA positive	Favors epithelial NET/NEC.
Is it paraganglioma?	Keratins negative, S100/SOX10+ sustentacular pattern, SDHB loss (not contributory if retained), GATA3/PHOX2B positive	Favors paraganglioma.
Is it medullary thyroid carcinoma?	Calcitonin, CEA, TTF-1, PAX8 may overlap; thyroid correlation needed	Laryngeal NENs can be calcitonin-positive, so calcitonin alone does not prove MTC.
Is it squamous cell carcinoma/MiNEN?	p63/p40/CK5/6 on the non-neuroendocrine component	Helps identify mixed tumors or exclude pure NEC.
Is it high-grade NEC?	Ki-67 high, p53 aberrant, Rb loss	Supports small-cell/large-cell NEC biology.

MiNEN—mixed neuroendocrine–non-neuroendocrine neoplasm; MTC—medullary thyroid carcinoma; NEC—neuroendocrine carcinoma; NEN—neuroendocrine neoplasm; NET—neuroendocrine tumor.

**Table 5:** Review of the literature of laryngeal MiNEN.

No.	Publication, Year	Sex/ Age	Non-Neuroendocrine Compoennt	Neuroendocrine Compoennt	Metastasis	Therapy	Follow-Up (Months)
1	Eusebi et al. 1978 [88]	M/83	SCC	SmCC	Lymph nodes	S	DOD (24)
2	Gnepp et al. 1983 [104]	M/57	SCC	SmCC	Lymph nodes, bone	S + R	DOD (3.5)
3	Mills et al. 1983 [105]	M/49	SCC	SmCC	Lymph nodes	S + R	AFD (6)
4	Ferlito et al. 1985 [89]	M/50	SCC	SmCC	Lymph nodes	S + R	DOD (14)
5		M/54	SCC	SmCC	No	S + R	AFD (42)
6		M/47	SCC	SmCC	Lymph nodes, bone, brain, lung	R + C	DOD (48)
7		M/45	SCC	SmCC	Lymph nodes	R + C	DOD (21)
8		M/56	SCC	SmCC	No	R + C	AFD (77)
9	Chen et al. 1986 [90]	M/56	SCC	SmCC	No	S + C	AWD (13)
10		M/55	SCC	SmCC	Lymph nodes, bone, lung	S + R	DOD (9)
11	Cosby and Babin 1988 [91]	M/56	SCC	SmCC	Lymph nodes	S + C	NA
12	Gianoli et al. 1992 [92]	M/83	SCC	SmCC	No	S	AFD (8)
13	Yucel et al. 2000 [93]	M/32	SCC	SmCC	Lymph nodes	S + R + C	AWD (12)
14	Jaiswal and Hoang 2004 [94]	M/41	SCC	SmCC	No	R + C	AFD (8)
15	Barbeaux et al. 2006 [95]	M/61	SCC	SmCC	NA	R + C	AWD (44)
16		F/54	SCC	SmCC	Lymph nodes, bone, peritoneum, lung	R + C	AWD (36)
17	Davies-Husband et al. 2010 [96]	M/55	SCC	AC	Lymph nodes	C + R	AFD (12)
18	Aggarwal et al. 2010 [97]	M/59	SCC	SmCC	Lymph nodes	S + R	NA
19	Kołodziej et al. 2012 [98]	M/58	SCC	SmCC	NA	S	NA
20	Yu et al. 2019	M/61	SCC	SmCC	No	S + R + C	AFD (13)
21	Cox et al. 2022 [102]	M/59	SCC	SmCC	Lymph nodes	C	AWD (3)
22	Rajeshwari et al. 2020 [99]	M/50	SCC	SmCC	Lymph nodes	C + R	DOD (3)
23	Padilla-Cabello et al. 2021 [103]	M/67	SCC	SmCC	Lymph nodes	S + C	DOD (1)
24	Wang et al. 2022 [101]	F/54	Basaloid SCCC	LCNEC	Lymph nodes	S + R + C	AFD (12)
25	Hesse et al. 2023 [100]	M/64	SCC	LCNEC	Lymph nodes	C + R + I	AFD (30)

Cases predating the formal introduction of the term ‘MiNEN’ were included based on the original authors’ morphological description and immunohistochemical documentation of two distinct neuroendocrine and non-neuroendocrine components, which are retrospectively considered compatible with the current concept of MiNEN. M—male; F—female; LCNEC—large-cell neuroendocrine carcinoma; SCC—squamous-cell carcinoma; SmCC—small-cell carcinoma; AC—atypical carcinoid; NA—not available; S—surgery; C—chemotherapy; I—immunotherapy; R—radiotherapy; DOD—died of disease; AFD—alive free of disease; AWD—alive with disease.

## Data Availability

No new data were created or analyzed in this study. Data sharing is not applicable to this article.
